# Probiotics Blunt the Anti-Hypertensive Effect of Blueberry Feeding in Hypertensive Rats without Altering Hippuric Acid Production

**DOI:** 10.1371/journal.pone.0142036

**Published:** 2015-11-06

**Authors:** Cynthia Blanton, Zhengcheng He, Katherine T. Gottschall-Pass, Marva I. Sweeney

**Affiliations:** 1 Department of Nutrition, Idaho State University, Pocatello, Idaho, United States of America; 2 Department of Biology, University of Prince Edward Island, Charlottetown, Prince Edward Island, Canada; 3 Department of Applied Human Sciences, University of Prince Edward Island, Charlottetown, Prince Edward Island, Canada; Max-Delbrück Center for Molecular Medicine (MDC), GERMANY

## Abstract

Previously we showed that feeding polyphenol-rich wild blueberries to hypertensive rats lowered systolic blood pressure. Since probiotic bacteria produce bioactive metabolites from berry polyphenols that enhance the health benefits of berry consumption, we hypothesized that adding probiotics to a blueberry-enriched diet would augment the anti-hypertensive effects of blueberry consumption. Groups (*n* = 8) of male spontaneously hypertensive rats were fed one of four AIN ‘93G-based diets for 8 weeks: Control (CON); 3% freeze-dried wild blueberry (BB); 1% probiotic bacteria (PRO); or 3% BB + 1% PRO (BB+PRO). Blood pressure was measured at weeks 0, 2, 4, 6, and 8 by the tail-cuff method, and urine was collected at weeks 4 and 8 to determine markers of oxidative stress (F2-isoprostanes), nitric oxide synthesis (nitrites), and polyphenol metabolism (hippuric acid). Data were analyzed using mixed models ANOVA with repeated measures. Diet had a significant main effect on diastolic blood pressure (*p* = 0.046), with significantly lower measurements in the BB- vs. CON-fed rats (*p* = 0.035). Systolic blood pressure showed a similar but less pronounced response to diet (*p* = 0.220), again with the largest difference between the BB and CON groups. Absolute increase in blood pressure between weeks 0 and 8 tended to be smaller in the BB and PRO vs. CON and BB+PRO groups (systolic increase, *p* = 0.074; diastolic increase, *p* = 0.185). Diet had a significant main effect on hippuric acid excretion (*p*<0.0001), with 2- and ~1.5-fold higher levels at weeks 4 and 8, respectively, in the BB and BB+PRO vs. PRO and CON groups. Diet did not have a significant main effect on F2-isoprostane (*p* = 0.159) or nitrite excretion (*p* = 0.670). Our findings show that adding probiotics to a blueberry-enriched diet does not enhance and actually may impair the anti-hypertensive effect of blueberry consumption. However, probiotic bacteria are not interfering with blueberry polyphenol metabolism into hippuric acid.

## Introduction

Blueberry (BB) consumption confers protection against hypertension [1, 2], which affects 20–30% of adults in Canada [3] and the United States [4] and is a primary contributor to death by cardiovascular and cerebrovascular diseases [5, 6]. The health benefits of BB are attributable to its constituent polyphenols that act as potent antioxidants, vasorelaxants, and anti-inflammatory agents [7–11]. Our laboratory has previously demonstrated significant improvements in blood pressure (BP) and oxidative stress in spontaneously hypertensive rats fed a BB-enriched diet [7]. Identifying the specific BB compounds responsible for positive health outcomes is complicated by their transformation to multiple metabolites that can be difficult to characterize and quantify in biologic samples [12–14]. Recently, attention has been drawn towards the bioactivity of polyphenol metabolites produced by intestinal bacteria. The polyphenols not degraded by mammalian digestive enzymes pass to the colon, where bacteria metabolize them to secondary compounds that are absorbed into the body. These bacterial metabolites appear in the circulation at higher concentrations than the parent polyphenols and are thought to mediate many of the biologic benefits of BB and other polyphenol-rich foods [15–17]. This is an underexplored area of research.

The contribution of specific bacteria strains to polyphenol metabolism is not known. However, there is evidence supporting the *in vivo* fermentation of BB by probiotic bacteria. In both humans consuming a drink containing 25 g blueberry powder [18] and laboratory rats fed BB extract [19], intestinal *Bifidobacterium* and *Lactobacillus* numbers increased significantly. *In vitro* studies using cell cultures confirm that *Lactobacillus* and *Bifidobacterium* perform enzymatic reactions involved in the metabolism of polyphenols [20–22]. These findings suggest that the most commonly consumed probiotic bacteria produce secondary metabolites from berry phytochemicals. Furthermore, evidence indicates that the probiotics augment the health benefits of polyphenol-rich diets. In a rat model of colitis, feeding a combination of probiotic and BB husk enhanced anti-inflammatory outcomes better than either supplement alone [23]. This group of investigators also showed a synergistic effect of probiotics and rose hip in protecting against ischemia-reperfusion injury in mice [24]. In a mouse model of metabolic syndrome, dietary supplementation with a probiotic and green tea powder produced a greater reduction in inflammatory markers than green tea alone [25]. Probiotic consumption exerts independent beneficial effects on cardiovascular risk factors [26, 27], including blood pressure [28, 29], which could explain the additive and synergistic effects found in these studies.

Taken together, these findings suggest that probiotics may be useful as an adjuvant in enhancing the health outcomes of BB consumption. Specifically, probiotics could augment polyphenol-metabolizing microbiota populations and secondary metabolite production, which conceivably would improve the magnitude of dietary BB-related health outcomes. This report describes the effect of adding probiotics to a BB-enriched diet on blood pressure in an animal model of hypertension.

## Materials and Methods

### Animals and diets

This study was performed in strict accordance with the Guide to the Care and Use of Experimental Animals prepared by the Canadian Council on Animal Care and the Guide for the Care and Use of Laboratory Animals prepared by the National Institutes of Health. Use of animals and the study protocol were approved by the University of Prince Edward Island Animal Care Committee (protocol #14–026) and the Idaho State University Institutional Animal Care and Use Committee (protocol #721).

Thirty-two male spontaneously hypertensive rats (Charles River SHR/NCrl strain code 007, 8 weeks of age; 180–210 g body weight) were purchased from Charles River Laboratories (Quebec, Canada) [30]. Animals were housed individually in plastic cages with wood shaving bedding in a temperature-controlled room (21°C) with a 12-h light-dark cycle. The animal room was within the Specific Pathogen Free laboratory at the Atlantic Veterinary College at the University of Prince Edward Island (UPEI). Rats were acclimated to laboratory conditions and fed pellet chow for one week prior to beginning the experimental diets. Rat body weight and food consumption were measured weekly.

Groups of rats (*n* = 8) were randomly assigned to one of four diets: American Institute of Nutrition (AIN) ‘93G purified diet (control, CON); AIN-93G purified diet with 3% freeze-dried blueberry (BB); AIN-93G purified diet with 1% probiotic (PRO); and AIN-93G purified diet with 3% freeze-dried blueberry and 1% probiotic (BB+PRO). The composition of diets is described in **Table 1**. Freeze-dried BB powder was obtained from the Wild Blueberry Association of North America (Old Town, ME). BB powder was produced from samples of lowbush wild blueberries (vaccinium angustifolium) that grow in Maine and Canada. Fresh blueberries were frozen at the point of harvest, freeze-dried and stored at -20°C prior to incorporation into the rat diet. The probiotic used was VSL#3® (Sigma Tau Pharmaceuticals, Inc., Gaithersburg, MD; lot #8304L10, purchased online at www.vsl3.com) a mixture of eight live lyophilized bacteria: *Bifidobacterium breve*, *Bifidobacterium longum*, *Bifidobacterium infantis*, *Lactobacillus acidophilus*, *Lactobacillus plantarum*, *Lactobacillus paracasei*, *Lactobacillus bulgaricus and Streptococcus thermophiles*, with a total bacteria count listed on the label as 1.0x10^11^ colony-forming units (CFU)/g. VSL#3 was selected based on a meta-analysis showing significantly stronger effects of multi-strain vs. single-strain probiotic supplements on blood pressure [29]. A 1% VSL#3 concentration of the diet was chosen to achieve a dose demonstrated to produce significant anti-oxidative [31] and vascular effects in rats [32] within a daily food intake previously reported for young adult spontaneously hypertensive rats (15–20 g/d) [7, 33]. Powdered diets were prepared at UPEI using ingredients purchased from Dyets, Inc. (Bethlehem, PA). To make the BB- and probiotic-containing diets, these ingredients replaced the equivalent amount of cornstarch. All diets contained 2% NaCl (Table 1) to induce hypertension in the spontaneously hypertensive rats. All diets were stored at -20°C until the time of feeding and food cups were replenished daily with fresh diet. Viability of bacteria in the probiotic-containing diets was confirmed by analysis performed by the Microbiology Laboratory at Bio Food Tech, Charlottetown, PEI. Three samples of each diet were tested: Samples stored at -20°C; samples kept at animal-room temperature for 24 h; and samples kept at animal-room temperature for 48 h. Quantitative analysis showed ≥ 1.0x10^7^ CFU for each of aerobic and anaerobic bacteria per g PRO and BB+PRO diet.

**Table 1:** Composition of AIN’93G diets.

Ingredient^a^	Amount in diet (g/kg)			
	Control	Blueberry	Probiotic	Blueberry + Probiotic
Casein, high nitrogen, 80-mesh	200	200	200	200
Cornstarch	377.5	347.5	367.5	337.5
Dyetrose^b^	132	132	132	132
Sucrose	100	100	100	100
Cellulose	50	50	50	50
3% blueberry powder^a^ *	0	30	0	30
1% probiotic^a^	0	0	10	10
2% NaCl	20	20	20	20
Soybean oil + Tertiary Butyl Hydroquinone (0.056 g)	70	70	70	70
Mineral mix^c^	35	35	35	35
Vitamin mix^d^	10	10	10	10
L-Cystine	3	3	3	3
Choline bitartrate	2.5	2.5	2.5	2.5

^a^Ingredients purchased from Dyets, Inc. (Bethlehem, PA) except for blueberry powder (donated by the Wild Blueberry Association of North America) and probiotic (VSL#3® purchased from www.vsl3.com).

^b^Ninety percent tetrasaccharides and higher.

^c^Composition (g/kg mineral mix): CaCO_3_, 357.0; KH_2_PO_4_, 196.0; K Citrate•H_2_O, 70.78; NaCl, 74.0; K_2_SO_4_, 46.6; MgO, 24.3; Fe citrate, 6.06; ZnCO_3_, 1.65; MnCO_3_, 0.63; CuCO_3,_ 0.31; KIO_3_, 0.01; Na_2_SeO_4_, 0.01025; (NH_4_) _6_ Mo_7_O_24_•4H_2_O, 0.00795; Na_2_SiO_3_•9H_2_O, 1.45; CrK(SO_4_) _2_•12H_2_O, 0.275; LiCl, 0.0174; H_3_BO_3_, 0.0815; NaF, 0.0635; 2NiCO_3_•3Ni(OH) _2_•4H_2_O, 0.0318; NH_4_VO_3_, 0.0066.

^d^ Composition (g/kg vitamin mix): thiamin HCl, 0.6; riboflavin, 0.6; pyridoxine HCl, 0.7; nicotinic acid, 3.0; Ca pantothenate, 1.6; folic acid, 0.2; D-biotin, 0.02; vitamin B12 (0.1% in mannitol), 2.5; vitamin A palmitate (500 000 IU/g), 0.8; DL-α-tocopheryl acetate (500 IU/g), 15; vitamin D3 (400 000 IU/g), 0.25; vitamin K/dextrose 10 mg/g (phylloquinone), 7.5.

*Nutritional analysis of blueberry powder: 397 kcalories/100 g, 77.2% by weight available carbohydrate, 3.15% protein, 2.3% fat, 13.8% insoluble fiber, 3.6% soluble fiber.

### Blood pressure measurements

BP was measured at weeks 0, 2, 4, 6, and 8 using the CODA™ non-invasive BP system (Kent Scientific Corporation, Torrington, CT). CODA™ employs a volumetric blood flow/blood volume method to measure systolic and diastolic BP in the tail. Rats are individually placed in clear polycarbonate cylindrical holders designed with air holes, a nose cone, and a rear gate. The tail extends out of the rear gate through a hole, and to the tail are attached an occlusion cuff (at the tail base) and a Volume Pressure Recording cuff (distal to the occlusion cuff). The rat in the holder is placed on a warming platform to enhance tail blood flow, and the tail cuffs are attached via cords to a hardware controller unit. The user interface is a laptop computer installed with CODA™ software and connected to the controller unit. Rats were allowed to rest quietly on the warming platform for 15 min prior to BP measurements. During this time, body temperature was monitored to maintain body temperature at ~32°C using an infrared thermometer aimed at the tail base.

During each CODA™ session, BP was measured 15 times (5 initial acclimation measurements and 10 experimental measurements) over 8–10 min. Our protocol dictated that at least 5 of 10 experimental readings must be deemed valid by the CODA™ system to be considered a successful measurement set. If fewer than 5 valid measurements were obtained, a second set of 10 experimental measurements was collected immediately after the first set. For systolic and diastolic BP, the mean of 5 valid measurements was used in statistical analysis.

### Metabolic sample and tissue collection

At weeks 4 and 8, rats were placed individually in metabolic cages with access to water only for 16-h urine collections. Urine was stored in aliquots at −80°C until analysis.

At the end of the feeding period, rats were anesthetized deeply with sodium pentobarbital (70 mg/kg, intraperitoneal) and killed by exsanguination. Blood was collected into vials, allowed to coagulate at room temperature for 30 min, and then placed on ice prior to centrifugation at 1200 x g at 4°C for 15 min. Serum was divided into aliquots and stored at -80°C.

### Analysis of urine

Urine hippuric acid served as a biomarker of dietary polyphenol metabolism [34, 35] and was measured using the method described by Phipps [36]. Urine samples were centrifuged at 1750 x g at 22°C for 5 min and 150 μl of the supernatant were mixed with 150 μl pyridine and 60 μl benzenesulfonyl chloride. After 30 min, 1.5 ml ethanol were added and the samples were centrifuged at 1750 x g at 22°C for 5 min before reading in a spectrophotometer at a wavelength of 410 nm. Chemicals were purchased from Sigma-Aldrich (ON, Canada).

Urine F2-isoprostanes were used as markers of lipid peroxidation and oxidative stress. Urine samples were diluted 1:40 and F-2 isoprostane levels were measured using an enzyme-linked immunoassay kit (Cayman Chemical, Ann Arbor, MI). Urine nitrites were measured to estimate blood nitric oxide levels. Samples were processed using a Griess Reagent System kit (Promega Corporation, WI) and nitrite levels were measured by spectrophotometry at 540 nm. Urine hippuric acid and nitrites were normalized to urine creatinine concentrations. Creatinine was measured in urine samples diluted 1:10 using a colorimetric assay (Cayman Chemical, Ann Arbor, MI). The dilution factors were corrected for in all calculations.

### Statistical analyses

BP data distributions were analyzed and data not normally distributed (systolic BP and urine metabolites) were transformed. One-way mixed models analysis of variance (ANOVA) with repeated measures was used to analyze the effect of diet on systolic and diastolic BP, change (week 8 –baseline) in BP, and urine metabolites across time points. Post-hoc analysis was performed using Tukey’s adjustment for multiple comparisons. Results are reported as means ± standard error (SE) and differences were considered significant at *p*<0.05. Statistical Analysis Software (SAS) version 9.3 was used to perform analyses.

## Results

### Body weight, food consumption, and feed efficiency

There were no differences across groups in body weight at baseline (main effect of group, *p* = 0.80), or endpoint (week 8) (*p* = 0.84). Total body weight gain (*p* = 0.97), total food consumption (*p* = 0.91), and feed efficiency (*p* = 0.99) also were not different across groups (**Table 2)**.

**Table 2:** Body weight and food consumption.

Diet Group	Baseline Body Weight (g)	Endpoint Body Weight (g)	Body Weight gain (g)	Food consumption (g)consumption (g)	Feed efficiency (g Body Weight gain/g food consumption)
CON	199.1 ± 4.1	338.2 ± 5.5	139.1 ± 4.1	1030.6 ± 29.8	0.136 ± 0.007
BB	195.3 ± 2.1	332.9 ± 5.2	137.6 ± 5.4	1026.9 ± 52.9	0.136 ± 0.008
PRO	196.1 ± 2.9	331.8 ± 6.3	135.8 ± 6.8	1001.9 ± 42.7	0.138 ± 0.012
BB+PRO	197.6 ± 2.4	333.7 ± 5.2	136.0 ± 6.0	996.9 ± 33.0	0.137 ± 0.007
*P* value effect of diet within column	0.806	0.842	0.977	0.910	0.996

Baseline and endpoint (week 8) body weight, total body weight gain, total food consumption, and feed efficiency (body weight gain/food consumption) of rats fed control (CON), 3% blueberry (BB), 1% probiotic (PRO), or 3% blueberry + 1% probiotic (BB+PRO) diet for 8 weeks; *n* = 8 per diet group. Data are presented as means ± SEM, with statistical analysis performed using mixed models ANOVA. There was no statistically significant main effect of diet on any parameter **(S1 Dataset)**.

### Blood pressure response to diet

**Fig 1:**
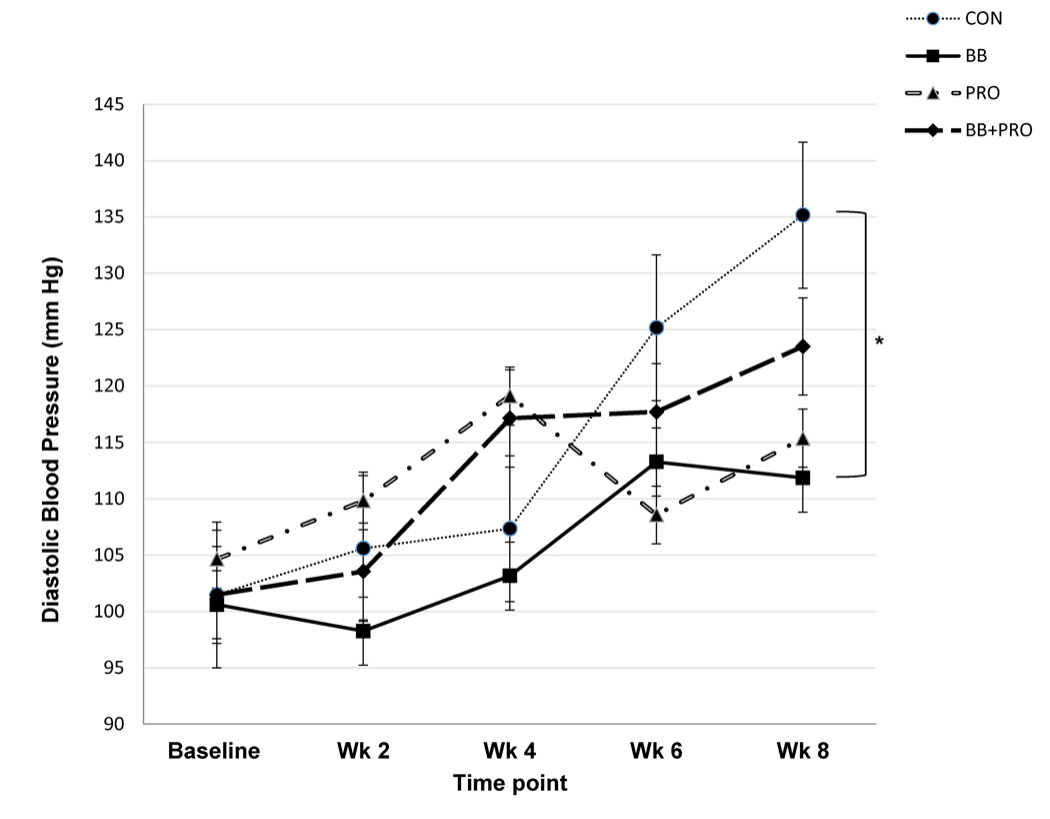
Diastolic blood pressure. Diastolic blood pressure of spontaneously hypertensive rats fed control (CON), 3% blueberry (BB), 1% probiotic (PRO), or 3% blueberry + 1% probiotic (BB+PRO) diet for 8 weeks. Data points are presented as means ± SEM for 8 rats in each diet group, with statistical analysis performed by mixed models ANOVA with repeated measures and post-hoc comparisons with Tukey’s adjustment. Diet exerted a significant main effect on diastolic blood pressure (*p* = 0.046). Asterisk (*) indicates statistically significant difference between BB and CON rats across weeks 2–8 (*p = 0.035). Within-week post-hoc comparisons between BB and CON rats showed no statistically significant differences when using Tukey’s adjustment; unadjusted p value for BB vs. CON at week 8 was *p* = 0.003 (S2 Dataset).

**Fig 2:**
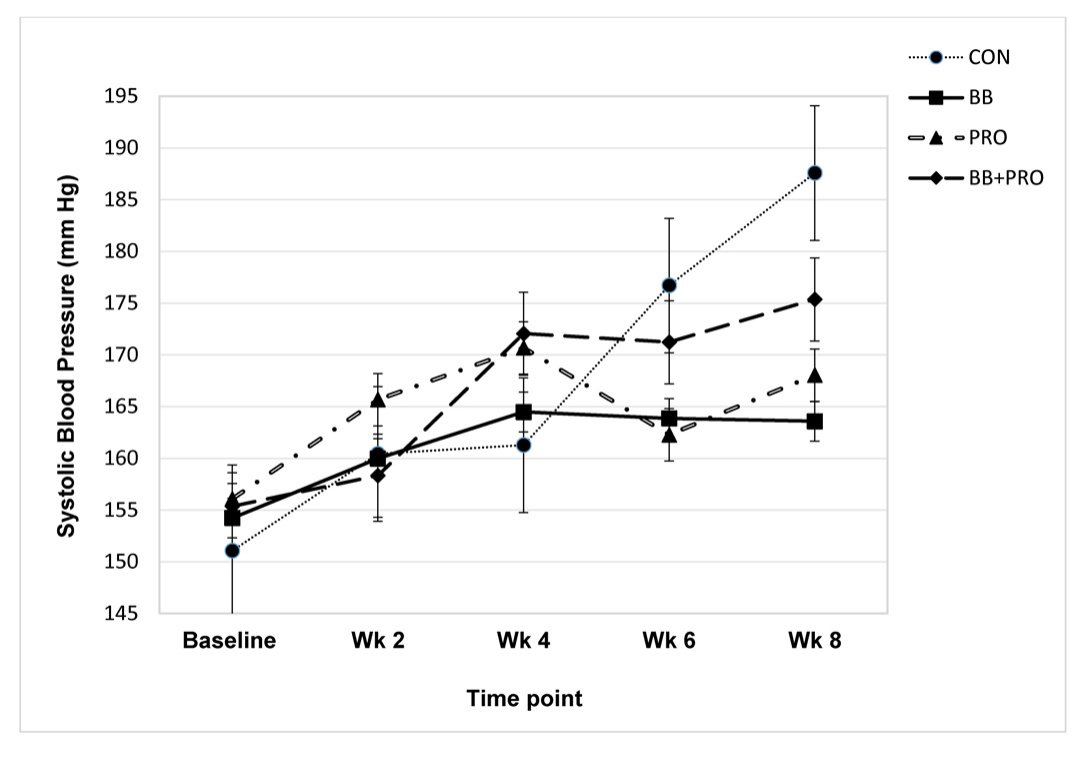
Systolic blood pressure. Systolic blood pressure of spontaneously hypertensive rats fed control (CON), 3% blueberry (BB), 1% probiotic (PRO), or 3% blueberry + 1% probiotic (BB+PRO) diet for 8 weeks. Data points are presented as means ± SEM for 8 rats in each diet group, with statistical analysis performed by mixed models ANOVA with repeated measures. Diet did not exert a significant main effect on systolic blood pressure across time points (*p* = 0.220) (S2 Dataset).

Diet had a significant main effect on diastolic BP across the experiment (*p* = 0.046), with significantly lower measurements in the BB-fed compared to CON-fed rats (post-hoc analysis with Tukey’s adjustment, *p* = 0.035, **Fig 1)**. Systolic BP showed a similar but less pronounced response to diet (main effect of diet, *p* = 0.220), again with the largest difference occurring between the BB vs. CON groups (unadjusted *p* = 0.045; adjusted *p* = 0.187, **Fig 2)**.

BP increased from baseline over the 8-week study as hypertension intensified **(Figs 1 and 2)**. The absolute increases in BP from baseline to 8 weeks tended to be smaller in the BB and PRO vs. CON-fed rats (main effect of diet on increase in systolic BP, *p* = 0.074 and diastolic BP, *p* = 0.185, **Table 3)**. BB- and PRO-fed rats displayed 75% and 66%, respectively, smaller increases in systolic BP between baseline and week 8 compared to CON-fed rats. The rise in diastolic BP between baseline and week 8 was similarly attenuated (67% and 68% lower, respectively) by BB and PRO vs. CON feeding. This beneficial response was blunted in rats fed a combination of BB+PRO, in which increases in systolic and diastolic BP between baseline and week 8 were 45% and 55% lower, respectively, than CON-fed rats.

**Table 3:** Increase in blood pressure.

Diet Group	Increase in systolic blood pressure (mmHg)	Increase in diastolic blood pressure (mmHg)
CON	36.5 ± 9.9	33.6 ± 12.5
BB	9.3 ± 7.9*	11.2 ± 6.5
PRO	11.9 ± 6.5	10.7 ± 6.8
BB+PRO	20.0 ± 5.3	22.0 ± 5.3
P value effect of diet	0.074	0.185

Absolute increase in systolic blood pressure (SBP) and diastolic blood pressure (DBP) in mmHg from baseline to week 8 in spontaneously hypertensive rats fed control (CON), 3% blueberry (BB), 1% probiotic (PRO), or 3% blueberry + 1% probiotic (BB+PRO) diet; *n* = 8 per diet group. Data are presented as means ± SEM, with statistical analysis performed using mixed models ANOVA with post-hoc comparisons using Tukey’s adjustment. The main effect of diet on increase in SBP and DBP was not statistically significant.

Asterisk (*) indicates difference compared to CON for increase in SBP at *p* = 0.079 when using Tukey’s adjustment for multiple comparisons (unadjusted *p* = 0.018) **(S2 Dataset)**.

### Bioavailability of blueberry polyphenols

**Fig 3:**
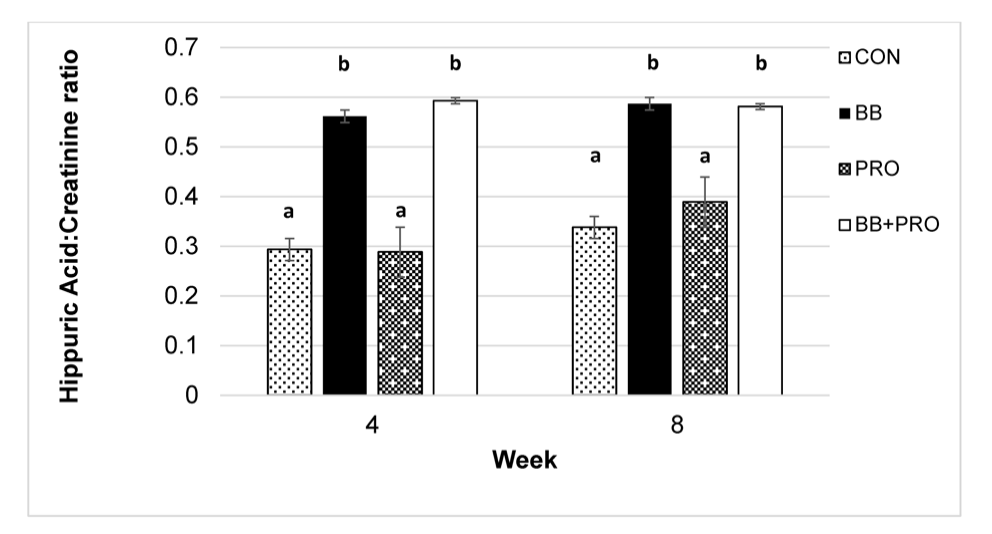
Urine levels of hippuric acid. Urine levels of hippuric acid normalized to creatinine at weeks 4 and 8 in spontaneously hypertensive rats fed control (CON), 3% blueberry (BB), 1% probiotic (PRO), or 3% blueberry + 1% probiotic (BB+PRO) diet for 8 weeks. Data points are presented as means ± SE bars for 8 rats in each diet group except CON week 8, where *n* = 7. Statistical analysis was performed using mixed models ANOVA with repeated measures and post-hoc comparisons with Tukey’s adjustment. Diet exerted a significant main effect on hippuric acid level, *p*<0.0001. Within week, means with different letters are significantly different at *p*<0.0001 except BB vs. PRO week 8 at *p* = 0.001 (S3 Dataset).

There was a significant main effect of diet on urine hippuric acid concentrations (p<0.0001), with the BB- and BB+PRO-fed rats excreting 2-fold and ~1.5-fold higher levels at weeks 4 and 8, respectively, than the CON- and PRO-fed rats (**Fig 3**)

### Biomarkers of oxidative stress and nitric oxide synthesis

**Fig 4:**
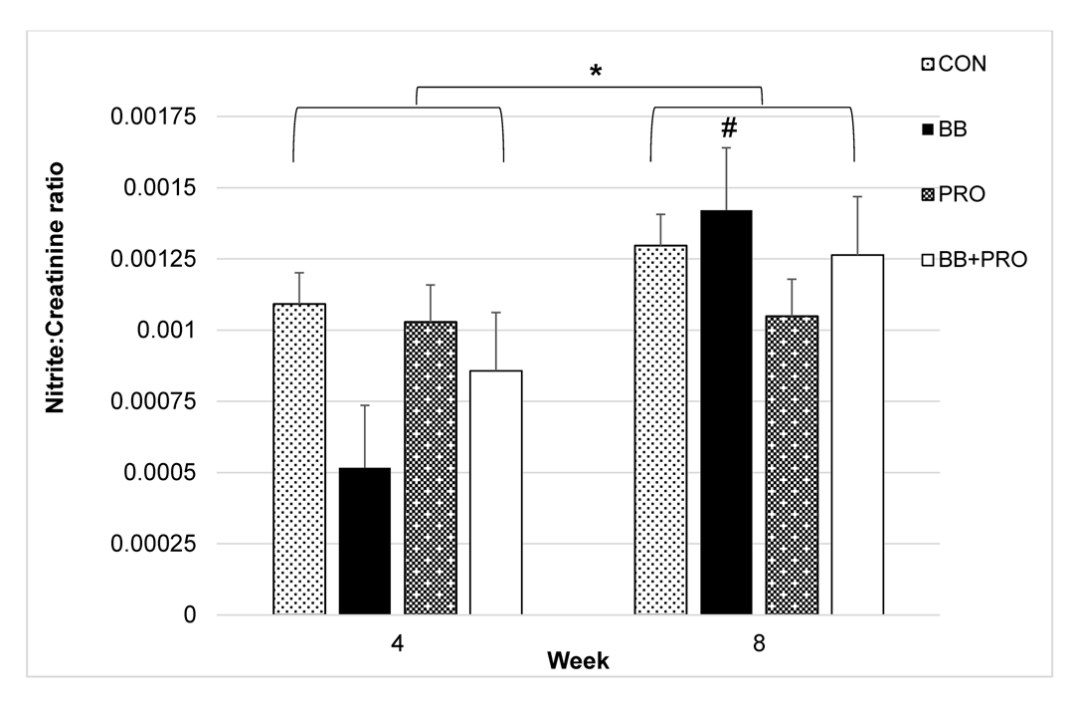
Urine levels of nitrite. Urine levels of nitrite normalized to creatinine at weeks 4 and 8 in spontaneously hypertensive rats fed control (CON), 3% blueberry (BB), 1% probiotic (PRO), or 3% blueberry + 1% probiotic (BB+PRO) for 8 weeks. Data points are presented as means ± SE bars for 8 rats in each diet group except CON week 8, where *n* = 7. Statistical analysis was performed using mixed models ANOVA with repeated measures and post-hoc comparisons with Tukey’s adjustment. There was no significant main effect of diet (*p* = 0.670). Asterisk (*) indicates statistically significant difference between week 4 and week 8 (*p = 0.009). Number sign (#) indicates difference between BB week 4 and BB week 8 at *p* = 0.07 (S4 Dataset).

Diet did not have a significant main effect on urine levels of F2-isoprostanes (*p* = 0.159) (S5 Dataset) or nitrites (*p* = 0.670). A significant effect of week was seen on nitrite levels (*p* = 0.009), driven by 2.7-fold and 1.9-fold increases in the BB- and BB+PRO-fed rats, respectively, from week 4 to week 8 (**Fig 4)**. The effect of an interaction between diet and week on nitrite levels was not significant (*p* = 0.267), and the increase in nitrites between weeks 4 and 8 in the BB-fed rats did not reach statistical significance (*p* = 0.070).

## Discussion

This study tested the hypothesis that probiotic supplementation enhances the BP-lowering effects of a BB-enriched diet. We reasoned that since probiotic bacteria metabolize polyphenols to bioactive compounds, adding probiotics to a polyphenol-rich diet might increase the production of anti-hypertensive metabolites, resulting in a greater reduction in blood pressure than polyphenol enrichment alone. Our findings do not support this hypothesis. To the contrary, supplemental probiotics reduced the beneficial BP effect of BB consumption in spontaneously hypertensive rats. While BB-fed rats showed a 75% reduction in systolic BP rise from baseline to endpoint compared to CON-fed rats, BB+PRO feeding resulted in only a 45% reduction. There are several points to consider in interpreting these findings.

First, probiotics did not interfere with the bioavailability of the BB polyphenols that are metabolized to hippuric acid. Urine hippuric acid concentration is a marker of polyphenol absorption [34, 37], and levels were significantly higher in the BB and BB+PRO groups vs. CON and PRO groups. Other polyphenol metabolites were not measured, however, and probiotic supplementation could have affected their production and biologic effects. BB feeding causes significant increases in plasma, urine, and fecal anthocyanin (a class of polyphenols) levels [34], and differences in concentrations of these compounds between BB- and BB+PRO-fed rats might have contributed to differences in BP outcomes. Nonetheless, our data suggest that probiotic metabolites of BB polyphenols are not responsible for the BP-lowering actions of BB-containing diets.

Second, freeze-dried BB did not reduce bacteria viability in the BB+PRO diet, as shown by similar bacterial CFU counts in the PRO and BB+PRO diets. This is evidence that the BB+PRO- and PRO-fed rats received similar probiotic doses and that differences in BP between these groups were not a result of probiotic destruction by BB coexistent in the diet. It is possible, however, that adding BB to the PRO diet altered the bacterial strain composition of the PRO diet. This speculation is based on previous findings of different microbiota populations in rats fed probiotics vs. probiotics and polyphenol-rich rose hips [24] or bilberry [38]. In the current study, PRO-fed rats demonstrated a reduction in blood pressure compared to controls, a finding consistent with the literature [29]. The partial negation of this effect in BB+PRO-fed rats might be attributable to differences in intestinal microbiota composition induced by the PRO vs. BB+PRO diets. The interactions between probiotics, commensal bacteria, and dietary polyphenols in the gastrointestinal tract are complex and not fully understood [39–41]. A beneficial shift in microbiota composition in PRO-fed rats, which might involve correcting hypertension-related dysbiosis [42, 43] and/or dyslipidemia [44–46], could have been disrupted in BB+PRO-fed rats. Although evidence supports that growth of *Lactobacillus* and *Bifidobacterium* (probiotics used in the current study) is enhanced by polyphenols *in vitro* and *in vivo* [47–51], and that co-ingestion of probiotics and polyphenols synergistically reduces inflammation [23–25], our testing of a BB+PRO diet on blood pressure response did not improve upon a BB diet. Genetic profiling of intestinal microbiota and host metabolomics analysis in BB-, PRO- and BB+PRO-fed rats would clarify the mechanisms underlying the different BP responses in the current study.

Nitric oxide synthesis, assessed by urine nitrite concentration, tended to increase between weeks 4 and 8 in response to BB feeding. Nitric oxide (NO) is a vasodilator important in BP regulation [52–54] and dysregulated NO activity is associated with hypertension in humans and rats [55, 56]. BB-enriched diets increase aortic wall vasorelaxation [10, 57] and reduce BP in hypertensive rats [7, 58] and humans [1, 59, 60]. Further, BB extract confers antioxidant protection in association with endothelial nitric oxide synthase (eNOS) induction in rat hearts exposed to reperfusion injury [61]. In women, reduced systolic and diastolic BP resulting from BB consumption were associated with increased NO production [60]. In the current study, BB-fed rats also showed a coincident rise in nitrite production and fall in BP. Interestingly, this nitrite response was blunted in BB+PRO-fed rats. It might have been expected that nitrite levels in the BB+PRO group would closely resemble those in the BB group, considering the groups’ similar increases in hippuric acid excretion (an indicator of polyphenol absorption). On the other hand, evidence supports an independent effect of probiotics on nitric oxide synthesis, which could account for the change in nitrite production when probiotics were added to the BB diet. Probiotic bacteria, specifically *Lactobacillus* and *Bifidobacterium* strains, stimulate nitric oxide production in macrophages [62], and VSL#3 (the probiotic mixture used in the current study) supplementation modulates eNOS activity in rats with portal hypertension [32]. Therefore, it is possible that the differences in BP response in the BB vs BB+PRO groups involve NO pathways; however, the lack of significant effects of diet on nitrite levels in this study suggest the contribution of other mechanisms.

Urinary F2-isoprostane levels were not affected by diet, suggesting that change in systemic oxidative stress was not a significant modulator of BP outcomes in this study. These findings are consistent with those from a previous study in our laboratory that showed BB feeding lowered BP but not urine F2-isoprostane levels in stroke-prone spontaneously hypertensive rats [7]. In that study we found less proteinuria (indicating less kidney damage) and reduced kidney nitrite concentrations (suggesting greater oxygen radical sequestration) in BB-fed hypertensive rats. This led us to postulate that BB-associated protection against oxidative damage is exerted at the level of the kidney, and thus may not be detectable using systemic markers of oxidative stress. In support of this explanation are published findings that renal oxidative stress, measured by kidney tissue F2-isoprostane concentration, is elevated in hypertensive rats [63], and administration of an antioxidant prevents BP rise and kidney damage in hypertensive rats [64, 65]. The impact of BB feeding on oxidative stress within the kidney remains a promising direction of investigation, particularly in light of studies showing reduced kidney oxidative stress after BB feeding in a rat models of hypertension and metabolic syndrome [58, 66].

In conclusion, addition of probiotics to a BB-enriched diet did not enhance the anti-hypertensive effects of the BB diet. The blunted blood pressure response of BB+PRO-fed vs. BB-fed rats appears not to result from differences in BB polyphenol absorption, nitric oxide production, or systemic oxidative stress.

## Supporting Information

S1 Dataset Feed efficiency. (XLSX)

S2 Dataset Blood Pressure. (XLSX)

S3 Dataset Hippuric Acid. (XLSX)

S4 Dataset Nitrites. (XLSX)

S5 Dataset Isoprostanes. (XLSX)
